# Epigenetic age of the pre-frontal cortex is associated with neuritic plaques, amyloid load, and Alzheimer’s disease related cognitive functioning

**DOI:** 10.18632/aging.100864

**Published:** 2015-12-18

**Authors:** Morgan E. Levine, Ake T. Lu, David A. Bennett, Steve Horvath

**Affiliations:** ^1^ Human Genetics, David Geffen School of Medicine, University of California Los Angeles, Los Angeles, CA 90095, USA; ^2^ Center for Neurobehavioral Genetics, University of California Los Angeles, Los Angeles, CA 90095, USA; ^3^ Rush Alzheimer’s Disease Center, Rush University Medical Center, Chicago, IL 60612, USA; ^4^ Department of Neurological Sciences, Rush University Medical Center, Chicago, IL 60612, USA; ^5^ Biostatistics, School of Public Health, University of California Los Angeles, Los Angeles, CA 90095, USA

**Keywords:** epigenetics, neuritic plaques, amyloids, cognitive functioning, memory, Alzheimer's disease, epigenetic clock, DNA methylation

## Abstract

There is an urgent need to develop molecular biomarkers of brain age in order to advance our understanding of age related neurodegeneration. Recently, we developed a highly accurate epigenetic biomarker of tissue age (known as epigenetic clock) which is based on DNA methylation levels. Here we use n=700 dorsolateral prefrontal cortex (DLPFC) samples from Caucasian subjects of the Religious Order Study and the Rush Memory and Aging Project to examine the association between epigenetic age and Alzheimer’s disease (AD) related cognitive decline, and AD related neuropathological markers.

Epigenetic age acceleration of DLPFC is correlated with several neuropathological measurements including diffuse plaques (r=0.12, p=0.0015), neuritic plaques (r=0.11, p=0.0036), and amyloid load (r=0.091, p=0.016). Further, it is associated with a decline in global cognitive functioning (β=−0.500, p=0.009), episodic memory (β=−0.411, p=0.009) and working memory (β=−0.405, p=0.011) among individuals with AD. The neuropathological markers may mediate the association between epigenetic age and cognitive decline. Genetic complex trait analysis (GCTA) revealed that epigenetic age acceleration is heritable (h^2^=0.41) and has significant genetic correlations with diffuse plaques (r=0.24, p=0.010) and possibly working memory (r=−0.35, p=0.065). Overall, these results suggest that the epigenetic clock may lend itself as a molecular biomarker of brain age.

## INTRODUCTION

Cognitive aging is on a continuum from normality, to mild cognitive impairment (MCI), to dementia [1–3]. Aging is also tied to an increasing susceptibility for a number of neurodegenerative diseases. After the age of 65 the risk of developing a neurodegenerative form of dementia, such as Alzheimer’s Disease (AD), has been shown to double every five years, and by age 85, the prevalence of dementia is estimated to be as high as 31% [4].

AD dementia is an irreversible progressive neurodegenerative disease affecting the central nervous system. It is typically characterized by the presence of amyloid-beta plaques and hyperphosphorylated paired helical filament tau protein–rich neurofibrillary tangles (NFT) [5]. Both types of lesions have been linked to AD dementia, MCI, and cognitive decline. There is also evidence that NFT mediates the association between amyloid plaques and clinical manifestations of AD [6]. While the exact physiology through which NFT and amyloid-beta plaques influence AD pathogenesis remains somewhat unclear, the presence of such deposits among those afflicted with AD is typically associated with much steeper trajectories of cognitive deficit accumulation with age [7, 8]. Cognition is not a unitary process but is com-posed of several dissociable cognitive systems, such as episodic memory the clinical hallmark of AD dementia.

Epigenetic alterations, such as DNA methylation (DNAm), have been linked to the both AD pathology [9] and cognitive aging in the absence of AD dementia [10]. DNAm refers to the addition of a methyl group to a cytosine nucleotide at cytosine-phosphate-guanine (CpG) sites. Hyper- or hypo methylation of sites can change over time, as a function of genes and environment, and have implications for gene expression via alterations in chromatin structure. We recently developed a highly accurate molecular biomarker of aging based on DNA methylation (DNAm) levels [11], known as “epigenetic clock”, which can be used to measure the age of human cells, tissues, and organs. Given that aging is associated with a normal loss in cognitive ability as well as the rapidly increasing susceptibility to AD, an aging biomarker based on DNAm could account for between-person differences in either the rate of cognitive aging among non-demented individuals or the rate of disease progression among those with AD. As a result, the goals of our study were to 1) examine the association between DNAm age and AD neuropathology, 2) test whether DNAm age relates to AD dementia status and measures of cognitive functioning, 3) determine if differences in DNAm age reflect cognitive decline in persons with or with AD-dementia, 4) examine whether neuropathology underlies the association between higher DNAm age and worse cognitive functioning. We hypothesize that participants who have higher levels of neuropathology, lower cognitive functioning, and/or who are diagnosed with AD will have higher DNAm age in PFC samples at death—signifying that their brains are biologically older. We also hypothesize that neuropathology will mediate the association between DNAm age and cognition.

## RESULTS

### Study Sample

Our analytic sample included Caucasian subjects from the Religious Order Study (ROS) and the Rush Memory and Aging Project (MAP) [12, 13]. Both are longitudinal community based cohort studies of aging and dementia. The majority of participants in both studies are 75–80 years old at baseline with no known dementia. All participants agree to organ donation at death. Participants sign and informed consent, repository consent, and Anatomical Gift Act. The studies were approved by the Institutional Review Board of Rush University Medical Center. Inclusion in the studies requires participants to consent to undergoing annual clinical evaluations as well as postmortem organ donation. The ROS sample includes Catholic priests, nuns, and brothers from across the United States, whereas the MAP sample includes a more general community based population from northeastern Illinois. For our analysis, we excluded subjects with missing DNAm age, or who were diagnosed with dementias other than AD leaving us with 700 Caucasian subjects. Participants were administered annual structured interviews and a battery of cognitive tests such as episodic memory (EM), working memory (WM), and semantic memory (SM), perceptual orientation (PO), and perceptual speed (PS). Tests were averaged to yield a measure of global cognitive functioning (GCF). Neuropathological assessments were carried out postmortem as described in Methods.

### Sample characteristics

As shown in Table 1, upon enrollment into the two studies, subjects were 63–102 years of age (mean= 81.36, standard deviation=6.59). Cognitive follow-up time after baseline ranged from 0 to 16 years, with a mean of 4.07 years (s.d.=3.42). Of 700 participants, 615 had at least three measures of cognitive functioning (baseline plus two follow-up), while half of our participants had seven or more cognitive measures. Average lifespan was approximately 89 years (s.d.=6.44). Overall, subjects from ROS (n=375) were 5 years younger at baseline and lived 1.5 years longer compared to those from MAP (n=325). Nearly two-thirds of participants (63.6%) were female.

**Table 1:** Sample characteristics

Variable	Statistic
Age at Enrollment, Mean (Std. Dev.)	81.4 (6.95)
Age at Death, Mean (Std. Dev.)	88.1 (6.60)
DNAm Age, Mean (Std. Dev.)	66.2 (5.04)
GCF, Mean (Std. Dev.)	−0.33 (0.90)
EM, Mean (Std. Dev.)	−0.28 (1.08)
WM, Mean (Std. Dev.)	−0.23 (0.90)
SM, Mean (Std. Dev.)	−0.31 (0.99)
PO, Mean (Std. Dev.)	−0.34 (0.92)
PS, Mean (Std. Dev.)	−0.53 (1.06)
Amyloid Load, Mean (Std. Dev.)	3.47 (3.68)
NP, Mean (Std. Dev.)	0.80 (0.84)
DP, Mean (Std. Dev.)	0.71 (0.80)
NFT, Mean (Std. Dev.)	0.60 (0.77)
Tangle Score, Mean (Std. Dev.)	6.52 (8.16)
Sex (Female=1), Frequency	0.636
Study (ROS=1), Frequency	0.536
AD Status, Frequency	0.433

Just over 300 of our 700 participants were diagnosed with AD dementia. Mean GCF, EM, WM, SM, PO, and PS were -0.33 (s.d.=0.90), -0.28 (s.d.=1.08),-0.23 (s.d.=0.90), -0.31 (s.d.=0.99), -0.34 (s.d.=0.92), and -0.53 (s.d.=1.06), respectively. Additionally, between- and within-person standard deviations were 0.82 and 0.47 for GCF, respectively; 1.00 and 0.55 for EM, respectively; 0.77 and 0.53 for WM, respectively; 0.91 and 0.53 for SM, respectively; 0.83 and 0.52 for PO, respectively; and 0.95 and 0.59 for PS, respectively. Finally, mean overall amyloid level was 3.47 (s.d.=3.68), mean neuritic plaque average 0.80 (s.d.=0.84), mean diffuse plaque average 0.71 (s.d.=0.80), mean NFT (silverstain) average 0.60 (s.d.=0.77), and mean overall paired helical filament (PHF) tangle score 6.52 (s.d.=8.16).

### Epigenetic age relates to neuropathological variables

**Figure 1:**
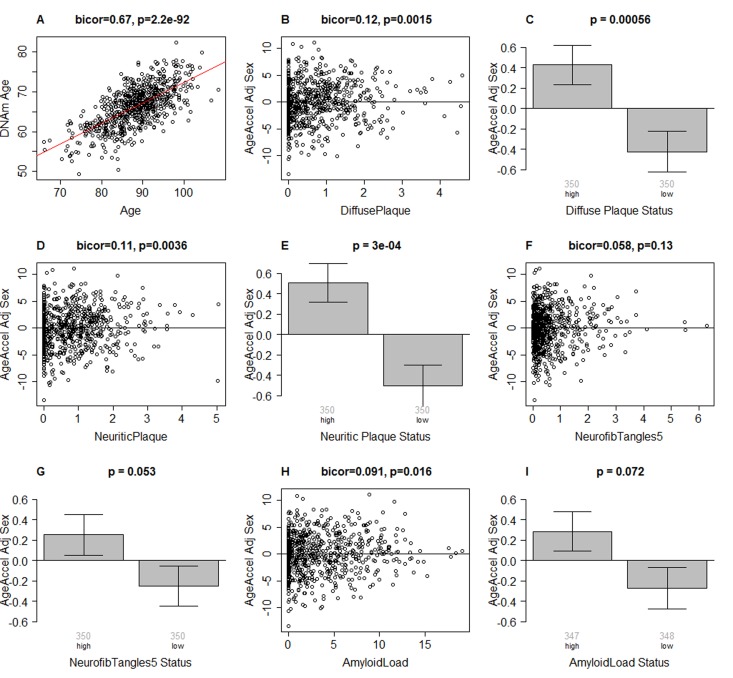
Epigenetic age of DLPFC samples versus neuropathological measures (**A**) Scatter plot relating the DNAm age of each PFC sample (y-axis) versus chronological age at time of death (x-axis). The red line depicts a linear regression line. The y-axis of the remaining panels (**B**-**I**) involves the measure of epigenetic age acceleration which has been adjusted for sex. The scatter plots relate epigenetic age acceleration (y-axis) to (**B**) diffuse plaques, (**D**) neuritic plaques, (**F**) NFTs, and (**H**) amyloid load. The title of each scatter plot reports a robust correlation coefficient (biweight midcorrelation) and a corresponding p-value. (**C,E,G,I**) The x-axis of the bar plots involve a binary grouping variable that results from using the median value for dichotomizing (**C**) diffuse plaques, (**E**) neuritic plaques, (**G**) NFT, and (**I**) beta-amyloid load, respectively. Each bar plot depicts the mean value, one standard error, and reports the p-value results from a non-parametric group comparison test (Kruskal Wallis test). The title of each scatter plot reports a robust correlation coefficient (biweight midcorrelation) and a corresponding p-value.

We estimated the epigenetic age (also known as DNAm age) of each brain samples by averaging the DNAm levels of 353 CpGs as described in Methods and [11]. DNAm age (in units of years) estimates the number of years that passed since birth. DNAm age was highly correlated with chronological age at time of death across all samples (correlation r=0.67, Figure 1A). We defined a measure of epigenetic age acceleration as residual resulting from regressing DNAm age on chronological age and sex. Thus, a positive value of age acceleration indicates that the epigenetic age is higher than expected based on chronological age and sex. Our study addresses the hypothesis that epigenetic age acceleration (that measures deviations between DNAm age and chronological age) captures aspects of the biological age of brain tissue. We test this hypothesis by relating epigenetic age acceleration to various measures of neuropathology and cognitive functioning.

Results from biweight midcorrelation showed that epigenetic age acceleration is associated with several postmortem neuropathological indices. Epigenetic age acceleration had a correlation of 0.12 with diffuse plaques (p=0.0015, Figure 1B,C), 0.11 with neuritic plaques (p=0.0036, Figure 1D,E), and 0.019 with amyloid load (p=0.016, Figure 1H,I). Further, it showed a marginally significant association with neurofibrillary tangle status (p=0.053 in Figure 1G) when the latter was defined by dichotomizing the NFT variable by its median value. These associations were also examined using multivariate models (Table 2), adjusting for age at death, sex, and study (ROS vs MAP), and again, we found positive associations between DNAm age and neuritic plaques (β=0.45, p=0.004), diffuse plaques (β=0.47, p=0.004), amyloid load (β=0.10, p=0.006), NFT (β=0.38, p=0.021), and Tangle Score (β=0.03, p=0.041).

**Table 2:** Multivariate associations between DNAm age and neuropathological measures

	Beta Coefficient (One-Tailed P-Value)
Amyloid Load	0.100 (0.006)
NP	0.451 (0.004)
DP	0.468 (0.004)
NFT	0.377 (0.021)
Tangle Score	0.030 (0.041)

Results are from independent multivariate models that adjust for age at death, study, and sex

### DNAm age, cognitive functioning and AD status

As shown in Table 3, we used linear models, adjusting standard errors to account for multiple observations, to examine whether postmortem estimates of DNAm age were associated with GCF, EM, WM, SM, PO, PS, and/or AD status. We found associations between DNAm age and both GCF and EM, the clinical hallmark of AD. For instance, results showed that a one unit decrease in GCF was associated with about a one third of a year increase in DNAm age (β = −0.34, P = 0.019), while a one unit decrease in EM was also associated with about a one third of a year increase in DNAm age (β = −0.30, P = 0.009). By contrast, we did not find a relationship between DNAm age and WM (β = −0.16, P = 0.172), SM (β = −0.21, P = 0.072), PO (β = −0.10, P = 0.270), or PS (β = −0.13, P = 0.191). We also examined whether AD dementia status was associated with higher DNAm age. Results showed a moderate, but non-significant association (β = 0.38, P = 0.103).

**Table 3:** Associations between DNAm age and cognitive functioning, by AD status

	β (SE)	P-value
GCF	−0.340 (0.163)	0.019
EM	−0.297 (0.126)	0.009
WM	−0.160 (0.170)	0.172
SM	−0.205 (0.140)	0.072
PO	−0.102 (0.166)	0.270
PS	−0.134 (0.153)	0.191
AD Status	0.377 (0.298)	0.103

DNAm age was used as the dependent variable for all models. All models were run adjusting for study (ROS or MAP), age at death, age a clinical evaluation (accept for the model for AD), and sex. GCF=Global Cognitive Functioning, EM=Episodic Memory, WM=Working Memory, SM=Semantic Memory, PO=Perceptual Orientation, PS=Processing Speed. P-values represent significance assuming a one-tailed hypothesis test. Standard errors were adjusted via clustering by Sample ID, in order to account for multiple observations (except for the model for AD).

Using linear models, we then examined the association between DNAm age and cognitive functioning by AD dementia status (Table 4). Overall, we found no association between DNAm age and any of the cognitive functioning measures among participants without AD dementia which might reflect the relatively low variance of cognitive measures among controls. However, among participants with AD dementia, GCF, EM, and WM were all associated with DNAm age. Results showed that for persons who developed AD dementia, every one unit decrease in GCF was associated with a half a year increase in DNAm (β = −0.50, P = 0.009). Similarly, for persons who developed AD dementia, every one unit decrease in EM or WM was associated with about a 0.4 year increase in DNAm (EM: β = −0.41, P = 0.009; WM: β = −0.40, P = 0.011).

**Table 4:** Associations between DNAm age and cognitive functioning, by AD status

	Non-Demented Participants (n=397)			AD Participants (n=303)		
	β (SE)	P-value	β (SE)	P-value
GCF	−0.059 (0.503)	0.454	−0.500 (0.210)	0.009
EM	−0.209 (0.322)	0.258	−0.411 (0.173)	0.009
WM	0.340 (0.328)	0.836	−0.405 (0.177)	0.011
SM	−0.047 (0.429)	0.456	−0.262 (0.160)	0.051
PO	−0.049 (0.312)	0.437	−0.102 (0.210)	0.313
PS	−0.058 (0.304)	0.425	−0.178 (0.205)	0.193

DNAm age was used as the dependent variable for all models. All models were run adjusting for study (ROS or MAP), age at death, age a clinical evaluation, and sex. GCF=Global Cognitive Functioning, EM=Episodic Memory, WM=Working Memory. P-values represent significance assuming a one-tailed hypothesis test. Standard errors were adjusted via clustering by Sample ID, in order to account for multiple observations.

### Mediation analysis involving neuropathological

Using multivariate linear models, with DNAm age as the dependent variable and adjusting for study (ROS vs MAP), age at clinical assessment, age at death, and sex, we examined whether neuropathological measures accounted for the association between worse cognitive functioning (GCF, EM) and higher DNAm age (Table 5 and Table 6). All models were run on n=695 participants who had complete neuropathology data. Standard errors were adjusted to account for repeat cognitive measures. For each cognitive measure, seven models were run. The first model shows the association between the cognitive measure and DNAm age, after adjusting for covariates. We find that (as reported previously), GCF and EM were inversely associated with DNAm age (GCF: β=−0.336, p=0.020; EM: β=−0.286, p=0.012). Model 2, is similar to model 1, but includes the addition of amyloid load, to examine whether it alters the association between cognitive functioning and DNAm age. We find that amyloid load is significantly associated with DNAm age. Furthermore, it accounts for 31.8% and 30.8% of the association between DNAm age and GCF and EM, respectively. Model 3, is similar to model 1, but with the addition of neuritic plaques. We find that NP is significantly associated with DNAm age and accounts for 66.1% of the association between DNAm age and GCF, as well as 65.0% of the association between DNAm age and EM. Model 4, includes the addition of diffuse plaques, which is significantly associated with DNAm age. However, diffuse plaques only account for 15.5% of the association between DNAm age and GCF, and 17.8% of the association between DNAm age and EM. Model 5, includes the addition of neurofibrillary tangles, which is not significantly associated with DNAm age, yet NFT accounts for 25.9% of the association between DNAm age and GCF, and 23.4% of the association between DNAm age and EM. Model 6, includes the addition of overall tangle score, which, like NFT, is not significantly associated with DNAm age, yet it account for a significant proportion of the association between DNAm age and GCF (24.4%), as well as DNAm age and EM (19.9%). Finally, Model 7 is similar to model 1, but with the addition of all five neuropathology variables. We find that the inclusion of all these measures accounts for 52.4% of the association between DNAm age and GCF, and 51.4% of the association between DNAm age and EM.

**Table 5:** Neuropathological mediation of the association between GCF and DNAm age

	Beta Coefficient (One-Tailed P-Value)						
	Model1	Model2	Model3	Model4	Model5	Model6	Model7
GCF	−0.336 (0.020)	−0.229 (0.087)	−0.114 (0.256)	−0.284 (0.044)	−0.249 (0.088)	−0.254 (0.084)	−0.160 (0.193)
Amyloid		0.094 (0.015)					0.026 (0.305)
Neuritic Plaques			0.553 (0.004)				0.514 (0.025)
Diffuse Plaques				0.360 (0.044)			0.144 (0.268)
NFT					0.231 (0.139)		−0.028 (0.537)
Tangles						0.019 (0.165)	−0.016 (0.720)

**Table 6:** Neuropathological aediation of the association between EM and DNAm age

	Beta Coefficient (One-Tailed P-Value)						
	Model 1	Model 2	Model 3	Model 4	Model 5	Model 6	Model 7
EM	−0.286 (0.012)	−0.198 (0.064)	−0.100 (0.229)	−0.235 (0.032)	−0.219 (0.057)	−0.229 (0.048)	−0.139 (0.161)
Amyloid		0.094 (0.015)					0.028 (0.287)
Neuritic Plaques			0.538 (0.005)				0.487 (0.033)
Diffuse Plaques				0.368 (0.044)			0.165 (0.243)
NFT					0.210 (0.164)		−0.032 (0.540)
Tangles						0.016 (0.202)	−0.017 (0.725)

### Heritability and genetic correlation analysis

We estimated the heritability of epigenetic age acceleration using the GCTA software [14, 15] from SNP markers measured on the same subjects. We find that epigenetic age acceleration in DLPFC is highly heritable (h^2^=0.41, Table 7), which is similar to heritability estimate reported for blood [11, 16].

**Table 7:** Heritability analysis and genetic correlations

	Heritability		Genetic correlation with epigenetic age acceleration	
Trait (residuals)	Estimate	P	Estimate	P
DNAm age	0.41	0.19	—	—
Mean GCF	< 0.01	0.50	—	—
Mean WM	0.17	0.32	−0.19	0.12
Mean EM	< 0.01	0.50	—	—
Last GCF	< 0.01	0.50	—	—
Last WM	0.07	0.43	−0.35	0.065
Last EM	< 0.01	0.50	—	—
Amyloid	0.03	0.46	—	—
Neuritic plaque	0.05	0.43	0.78	0.014
Diffuse plaque	0.38	0.080	0.24	0.010
NFT	< 0.01	0.50	—	—
Tangles	< 0.01	0.50	—	—

The GCTA software was used to estimate the heritability (first two columns) and the genetic correlations with epigenetic age acceleration (last two columns).

We find that diffuse plaques are highly heritable (h^2^=0.38, Table 7) and have a significant genetic correlation with epigenetic age acceleration (r=0.24, p=0.010, Table 7). Neuritic plaques also exhibit a significant genetic correlation with epigenetic age acceleration (r=0.78, p=0.014) but the result needs to be interpreted with caution since neuritic plaques are at best weakly heritable (h^2^=0.05). We also find a suggestive genetic correlation with working memory at the last assessment (r=−0.35, p=0.065) but working memory is only weakly heritable (h^2^=0.07).

## DISCUSSION

Overall, we found that postmortem DNAm age in DLPFC was associated with neuropathological variables (Figure 1 and Table 2) and with pre-mortem measures of cognitive decline, after adjusting for chronological age, sex, and other possible confounders (Tables 3 and 4). Our mediation analysis (Tables 5–6) suggests that a proportion (up to 66%) of the association between DNAm age and measures of cognitive function is mediated by neuropathological measures. Our genetic analysis (Table 7) indicates that pleiotropic genetic loci affect epigenetic age acceleration, neuropathological variables, and cognitive traits.

The association between cognitive function and DNAm age is consistent with previous work showing that general cognitive ability—defined as a composite score for six cognitive function tests comprising working memory, non-verbal reasoning, constructional ability, and processing speed—was associated with DNAm age in pre-mortem blood samples [17]. However, previous work has not examined the role of neuropathology or AD dementia in the association between DNAm age and cognitive decline. Our study showed that about half of the association between DNAm age and cognition was accounted for by variations in neuropathological variables. For instance, we found that worse GCF and EM was associated with higher DNAm age; however, this association was significantly reduced or eliminated after adjusting for amyloid load or neuritic plaques.

Previous studies have shown that there is little or no age effect on many cognitive domains after accounting for common neuropathologies [18]. The extended preclinical phase of dementia is typically characterized by an accumulation of neuropathology underlying cognitive decline, and as such, pathologies have been shown to relate to decline across the entire continuum, from normal, to MCI, to dementia [19, 20].

Nevertheless, declines in cognitive functioning have been shown to be significantly steeper among those with AD [21–24]. In contrast to those with non-pathological cognitive aging, the more drastic cognitive decline associated with AD is thought to reflect AD-mediated neuronal injury, larger decreases in brain volume, functional disconnection between PFC and the hippocampus, and dramatic increases in ventricle size [3, 25].

While our results showed that DNAm age was associated with cognitive decline among persons with a clinical diagnosis of AD, we did not find an association between AD dementia status and DNAm age. One potential explanation for the lack of association between AD dementia status and DNAm age is that the clinical diagnosis AD is only an incomplete measure of the underlying neuropathology such as the accumulation of amyloid-beta plaques and NFT. For this reason, AD reflects a heterogeneous group, which is why the severity of AD (as estimated by cognitive decline or neuropathology) may be more strongly associated with DNAm age than AD dementia status alone. Among our participants, we found that both the between-person and within-person variance in GCF and EM change was much higher for those with AD versus those without AD dementia (Table 8), suggesting that 1) the AD group may be far more heterogeneous in regards to neurocognitive decline, and 2) experience far more cognitive aging changes. Additionally, this interpretation is supported by our analysis of neuropathological variables, for which we found strong associations between DNAm age and all five measures (amyloid load, neuritic plaques, diffuse plaques, NFT, and overall tangle score). Additionally, results from step-wise models showed that neuropathological variables, especially amyloid load and neuritic plaques, may explain the association between DNAm age and cognitive functioning. This suggests that increased DNAm age may influence changes in the regulation of amyloid proteins, contributing to AD neuropathology, and thus manifesting as steeper cognitive declines [26].

**Table 8:** Between- and within-person statistics for cognitive function, by AD

		No AD			AD		
		Mean	Std. Dev.	N	Mean	Std. Dev.	N
GCF	Overall	0.126	0.496	2554	−0.894	0.954	2105
	Between		0.448	397		0.786	300
	Within		0.235			0.649	
EM	Overall	0.252	0.649	2482	−0.928	1.139	2029
	Between		0.607	397		0.951	300
	Within		0.323			0.731	

**Figure 2:**
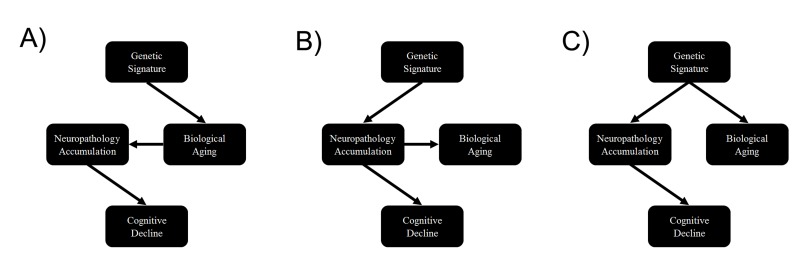
Causal scenarios that might explain the significant genetic correlations between epigenetic age, neuropathology and cognitive decline Genetic variants form a causal anchor that affect biological age (and associated measures such as epigenetic age) and various measures of neuropathology and cognitive decline.

Finally, our genetic analysis showed that DNAm age, neuropathology, and cognitive decline may be pleiotropic, which could reflect mediation among these factors. For instance, alleles could be associated with faster cognitive decline via acceleration of the biological aging process, which in turn leads to a faster accumulation of neuropathology (Figure 2A). Another alternative is that physiological consequences associated with neuropathological accumulation could influence both biological brain aging and cognitive decline, simultaneously (Figure 2B). Finally, loci could pleiotropically influence both neuropathology and biological aging, independently, with no causal pathway between them (Figure 2C). In moving forward, examination of these pathways will be important for facilitating our understanding of brain aging and neurodegenerative disease.

There are limitations to this study. First, DNAm age was only measured postmortem, which prevented us from determining if it was predictive of AD status or cognitive decline. Furthermore, DNAm age changes with time, yet in our sample it was only measured at a single time-point. For that reason we were unable to examine if larger changes in DLPFC DNAm age were associated with steeper cognitive decline or AD. Nevertheless, our study was strengthened by the inclusion of neuropathological variables, longitudinal measurements of multiple cognitive functioning domains, measurement of DNAm age in DLPFC rather than whole blood, and availability of postmortem data for neuropathologic indices.

Overall, our study shows that epigenetic aging in DLPFC is associated with the severity of cognitive decline as well as neuropathological hallmarks of AD. These results strongly suggests that the epigenetic clock lend itself as a molecular biomarker of brain age.

## METHODS

### Study sample

Our analytic sample included 700 non-Latino white subjects from the Religious Order Study (ROS) and the Rush Memory and Aging Project (MAP) [12, 13]. Both are longitudinal community based cohort studies of aging and dementia. The majority of participants in both studies are 75–80 years old at baseline with no known dementia. Inclusion in the studies requires participants to consent to undergoing annual clinical evaluations as well as postmortem organ donation. The ROS sample includes Catholic priests, nuns, and brothers from across the United States, whereas the MAP sample includes a more general community based population from northeastern Illinois. For our analysis, excluded subjects included those with missing DNAm age, or who were diagnosed with dementias other than AD.

### Clinical evaluations

Participants were administered annual structured interviews to assess cognitive functioning. These included tests for EM (immediate recall (word list), delayed recall (word list), word recognition (word list), immediate recall (East Boston story), delayed recall (East Boston story), logical memory immediate recall, logical memory delayed recall), WM (digits forward, digits backward, digit ordering), SM (Boston naming, category fluency, reading test), PO (line orientation, progressive matrices), and PS (symbol digits modality-oral, number comparison, stroop color naming, stroop color reading). For each domain, composite measures were calculated as the average across tests. Before taking the average, each cognitive test was converted to a z-score (with mean of zero and standard deviation of 1). Finally, GCF is meant to represent overall cognitive functioning. At each wave it was estimated as the average across z-scores from the 19 cognitive tests for EM, WM, SM, PO, and PS.

### Neuropathological examination

Upon participants’ death, brains were extracted, weighed, sectioned into 1 cm-thick coronal slabs, and stored. Neuropathological indices were examined in order to diagnose cognitive pathologies such as AD, Lewy Body diseases, and cerebrovascular disease [27]. Modified Bielschowsky silver stain was used to identify AD pathology based on NIA-Reagan and modified CERAD criteria. Global AD pathologic burden was estimated by averaging standardized numbers of neuritic plaques, diffuse plaques, and NFT across five brain regions as described in [28]. Moreover, amyloid load was quantified as abundance of amyloid-β, labeled with a N-terminal directed monoclonal antibody, while PHFtau tangles, was quantified as the density of paired helical filament tau tangles.

We focused on the following aggregated neuropathological variables (Figure 1 and elsewhere):

- ";neuritic plaques"; and ";diffuse plaques"; were defined as average of 5 scaled scores (namely scaled mid-frontal, temporal cortex, inferior parietal cortex, entorhinal cortex, and hippocampus plaques) [29, 30].
- ";NFT"; measures the tangle average across 5 regions (mid-frontal cortex, mid-temporal cortex, inferior parietal cortex, entorhinal cortex, and hippocampus CA1) [29].
- ";Amyloid load"; measures the overall amyloid load, which was defined as the mean amyloid scores across 8 regions (namely hippocampus entorhinal cortex, mid-frontal, inferior parietal cortex, anterior gyrus, calcarine cortex, cingulate regions, superior frontal gyrus) [29, 31, 32].
- Overall tangle score which reports the PHFtau tangle score across 8 regions (hippocampus, entorhinal cortex, midfrontal gyrus, inferior temporal, anterior gyrus, calcarine cortex. cingulate region, superior frontal gyrus).

### DNA methylation data

DNAm was measured using the Illumina Infinium HumanMethylation450 BeadChip. The Illumina BeadChips measures bisulfite-conversion-based, single-CpG resolution DNA methylation levels at 485577 different CpG sites in the human genome. These data were generated by following the standard protocol of Illumina methylation assays, which quantifies methylation levels by the β value using the ratio of intensities between methylated and un-methylated alleles. Specifically, the β value is calculated from the intensity of the methylated (M corresponding to signal A) and un-methylated (U corresponding to signal B) alleles, as the ratio of fluorescent signals β = Max(M,0) / [Max(M,0) + Max(U,0) + 100]. Thus, β values range from 0 (completely un-methylated) to 1 (completely methylated) (Dunning, 2008). The DNA methylation data are available at the following webpage https://www.synapse.org/#!Synapse:syn3168763. We focused on brain samples of Caucasian subjects from ROS and MAP that include brain donation at the time of death (n=700) [12, 13]. Additional details on the DNA methylation data can be found in [9].

### Epigenetic clock analysis and DNAm age

Several recent studies have proposed to measure the age of tissue samples by combining the DNA methylation levels of multiple dinucleotide markers, known as Cytosine phosphate Guanines or CpGs [11, 33, 34]. In particular, the epigenetic clock based on 353 Cytosine phosphate Guanine (CpG) markers was developed to measure the age (known as "DNA methylation age" or "epigenetic age") of human tissues, organs and cell types—including brain, breast, kidney, liver, lung, blood [11], and even applies to prenatal brain samples [35]. The epigenetic clock method - applied to two commercially standardized methylation platforms: the Illumina 450K array and the 27K arrays - is an attractive biomarker of aging because (1) it applies to most human tissues; (2) its accurate measurement of chronological age is unprecedented [11]; (3) it is predictive of all-cause mortality even after adjusting for a variety of known risk factors [16]; (4) it correlates with measures of cognitive and physical fitness in the elderly [17]; (5) it has already been useful in detecting accelerated aging due to obesity [36], Down syndrome [37], Parkinson’s disease [38], and HIV infection [39]; and . Further, the epigenetic clock was used to show that age acceleration of blood may predict the future onset of lung cancer [40], that the cerebellum ages slowly [41], that the blood of subjects with a severe developmental disorder ages normally [42], and that semi-supercentenarians and their offspring age more slowly [47].

Weighted DNAm measures across the 353 CpGs from the epigenetic clock were used to measure the DNAm age of DLPFC samples. These CpGs and their weights (coefficient values) were chosen in independent data sets by regressing chronological age on CpGs. DNAm age is then defined as predicted age, in years [43].

### Statistical analysis

Biweight midcorrelations and ordinary least squares regression models were used to examine whether postmortem neuropathology was associated with postmortem DNAm age in DLPFC, after controlling for age at death, study (ROS vs. MAP), and sex. For the bar plots in Figure 1, we defined a grouping variable (high versus low) by dichotomizing the respective neuropathological variable according to the median value. The median was chosen in order to arrive at equal group sizes (high versus low) and to avoid overfitting due to the selection of an optimal threshold. Multivariate linear regression models were fit in the whole sample and in strata defined by AD dementia status (according to the clinical diagnosis). Here we did not use a linear mixed effects model since our dependent variable (DNAm age) is a time-invariant variable based on postmortem brain tissue. The linear models were used to determine whether GCF, WM, EM, SM, PO, and PS over all waves leading up to death were related to postmortem DNAm age in DLPFC. Standard errors for cognitive decline models were adjusted to account for multiple observations. These models also included potential confounders, such as age of clinical evaluation, age at death, study (ROS vs. MAP), and sex. Finally, step-wise linear models were run with DNAm age as the dependent variable and cognitive measures as the independent models. For these models we examined how the association between DNAm age and cognition was altered with the inclusion of either one or all of the neuropathology measure.

We report one-sided (one-tailed) p-values for the cognitive scores and neuropathology variables in our multivariate model analyses because our hypotheses involving cognitive scores are one-sided (e.g. that *higher* DNAm age is associated with *worse* cognitive functioning and *higher* levels of neuropathology).

### Genetic analysis

Of the study samples, a total of 1102 individuals (632 normal/ 470 AD) were available with both genotypes and cognitive functioning or neuropathological measure. The GCTA software was used to estimate the heritability and genetic correlations based on both genotyped and imputed SNP markers. We used IMPUTE2 [44, 45] with haplotypes phased using SHAPEIT [46] to impute SNP and INDEL markers, with a reference panel based on the 1000 Genome haplotypes from 2,504 individuals (released in October 2014). As study individuals were genotyped on either Affymetrix SNP Array 6.0 or Illumina HumanOmniExpress, we performed imputation on each subset of individuals stratified by platform. We merged the imputation outputs across platforms and pruned in the markers with info measure > 0.4 in both sets. The other quality control was based on minor allele frequency (MAF) ≥ 0.02. We converted the IMPUTE2 output format to MaCH dosage format in order to use it as input for the GCTA software.
